# Determinants of adequate knowledge of postpartum warning signs and complications among parturients in Ibadan, Nigeria: a cross sectional study

**DOI:** 10.1186/s12884-025-08058-1

**Published:** 2025-08-28

**Authors:** Olaolu O. Oni, Oluwasomidoyin O. Bello, Anjola-Oluwa A. Ajayi

**Affiliations:** 1 Department of Obstetrics and Gynaecology, University College Hospital, Ibadan, Nigeria; 2 Department of Obstetrics and Gynaecology, College of Medicine, Faculty of Clinical Sciences, University of Ibadan, Ibadan, Nigeria

**Keywords:** Complications, Knowledge, Parturients, Postpartum, Warning signs

## Abstract

**Background:**

Postpartum maternal morbidity or mortality is one of the common unexpected outcomes of childbirth, hence postnatal care is critical to reducing it. This study assessed the parturients’ knowledge of postpartum warning signs and complications in a tertiary health facility at Ibadan, Nigeria.

**Methods:**

This was a cross-sectional study among 450 parturients using a semi-structured self-administered questionnaire to assess their knowledge of postpartum warning signs and complications. Data were analysed with SPSS version 25.0 using descriptive statistics and logistic regression with level of statistically significant set at *p* < 0.05.

**Results:**

A total of 450 parturients, with a mean age of 30.1 *±* 4.9 years participated. The most common complications known among the mothers were wound infection, 132 (29.3%), infection/sepsis, 116 (25.8%), episiotomy pain, 103 (22.9%), and hypertension, 102 (22.7%). Among the 180 parturients with the knowledge of warning signs, almost all 179 (99.4%) knew one warning sign, 136 (75.6%) knew two warning signs though only 21 (11.7%) knew five warning signs. About a third, 155 (34.4%) had adequate knowledge of postpartum complications. Determinants of adequate knowledge of postpartum complications were age (Odd ratio OR = 2.37, 95% CI = 1.107–5.057), educational status (OR = 0.37, 95% CI = 0.208–0.650), and duration of time used to relay the information by the healthcare providers (OR = 0.13, 95% CI = 0.041–0.425).

**Conclusions:**

The inadequate knowledge of the postpartum mothers about postpartum warning signs and complications is a cause for concern. Maternal age, higher educational attainment, and longer counselling duration were independently associated with adequate knowledge. To improve maternal knowledge and early recognition of complications, standardized and structured postpartum counselling should be institutionalized as part of routine postnatal care.

**Supplementary Information:**

The online version contains supplementary material available at 10.1186/s12884-025-08058-1.

## Background

Postpartum care of women and their infants provides an assessment of needs, available resources, and ongoing support to promote well-being, improve quality of life, and physical and mental health needs. The postpartum is a period of vulnerability when a woman must adapt to multiple physical, social, and psychological changes [1].

A maternal death occurred almost every 2 min, with 92% of these deaths occurring in developing countries [2]. Each day, over 700 women die from preventable pregnancy-related causes worldwide, with more than one-fifth of them occurring in Nigeria alone. In 2023, according to the World Health Organization, Nigeria recorded the highest maternal mortality ratio (MMR) globally with 993 deaths per 100,000 live births [2]. In comparison with other countries, MMRs in the United States, South Africa, and Chad were 17, 118, and 748 deaths per 100,000 live births, respectively [2]. These significant differences highlight the complex factors contributing to maternal mortality, especially in low-resource settings. Maternal mortality in resource-poor nations has been attributed to three delays: delay in deciding to seek care, delay in reaching health facility to seek care on time, and delay in receiving adequate treatment at the facility. Among all, the major cause of the first delay is a lack of awareness about obstetric danger or warning signs to decide to seek care among mothers [3]. Many of the postpartum complications leading to maternal morbidity arise during labour and delivery and in the first 1–2 weeks following delivery. Major acute obstetric morbidities include haemorrhage, sepsis, and pregnancy-related hypertension [4, 5].

Health improvement of mothers and infants as two vulnerable groups of the society has long been a global public health priority. This focus was initially highlighted in the Millennium Development Goals and was succeeded in 2015 by the Sustainable Development Goals (SDGs), with SDG 3.1 aiming to reduce the global MMR to less than 70 deaths per 100,000 live births by 2030 [6]. Notably, more than 50% of these cases have been reported during the postpartum period [7, 8]. While there has been a 40% reduction in maternal deaths globally between 2000 and 2023, progress remains insufficient and uneven, particularly in sub-Saharan Africa with Nigeria’s progress been slower at 13% during the same period [2]. Over 28.7% of all global maternal deaths occurred in Nigeria in 2023, representing a significant increase compared to earlier years [2]. Between 2005 and 2015, it is estimated that over 600,000 maternal deaths and no less than 900,000 maternal near-miss cases occurred in the country. A Nigerian woman has a 1 in 22 lifetime risk of dying during pregnancy, childbirth, postpartum or post-abortion, whereas in the most developed countries, the lifetime risk is 1 in 4,900 [9]. The common complications resulting in severe morbidities include postpartum haemorrhage, hypertensive disorders, and infections/sepsis which collectively contribute significantly to maternal mortality [10, 11]. Among these, postpartum haemorrhage is the most frequently reported complication and carries the highest case fatality rate, while infections and hypertensive complications such as eclampsia and preeclampsia remain prevalent contributors to severe maternal morbidity and mortality [5, 10, 11]. The burden of these conditions is further exacerbated by systemic challenges such as limited access to skilled birth attendants, inadequate infrastructure and resources, delays in seeking care, and low awareness of danger signs during the postpartum period [2, 5]. Women’s knowledge about danger signs such as severe vaginal bleeding, severe headache, preterm labour, rupture of membrane before the onset of labour, epigastric pain, severe abdominal pain, prolonged labour, convulsions, retained placenta, foul-smelling vaginal discharge, and fever of obstetric complications is profoundly essential to enhance the utilization of skilled care during delivery and to seek emergency obstetric services [12]. Healthcare providers emphasise these warning signs during antenatal care, intrapartum care (skilled birth attendance at delivery), postpartum care, and emergency obstetric services, all of which are part of the pillars of safe motherhood initiatives aimed at improving maternal and neonatal outcomes. However, when women lack awareness of the warning signs of complications during pregnancy, childbirth, and the postpartum period, their ability to fully engage with these life-saving interventions is significantly limited [12, 13]. Studies have shown that only 15.6–37.3% of women are knowledgeable about warning signs during the postpartum period, with the level of knowledge significantly associated with factors such as maternal age, level of education, multiparity, number of antenatal visits, number of live births, and place of last delivery [12–16]. These findings reflect persistent gaps in awareness that contribute to delays in seeking skilled care, particularly in the postpartum period, and ultimately impact maternal health outcomes. Educating parturient on the warning signs can be a cost-efficient way of reducing maternal morbidities and mortalities [17].

In Nigeria, where a high incidence of postpartum morbidity exists, it is hoped that counselling, parturient good knowledge of warning signs and complications, and supervision of deliveries by skilled medical personnel will reduce this incidence [4]. So, there is a need for new mothers during their antenatal care visits or hospital stay to have received consistent messages about potential postpartum warning signs and complications that may ultimately reduce maternal death and the severity of maternal complications. Therefore, in view of this background, this study assessed the determinants of parturients’ adequate knowledge of postpartum warning signs and complications at a tertiary health facility in Ibadan, Nigeria.

## Methods

### Study design

This is a prospective cross-sectional study that determined the knowledge of postpartum warning signs and complications among postpartum mothers in a tertiary health facility in Ibadan, Southwestern Nigeria. The study was conducted from September 2021 to February 2022.

### Study location and population

The study was conducted at the University College Hospital, a tertiary health facility in Ibadan which is the largest city in West Africa. The hospital serves as a referral centre for private and public primary and secondary health facilities and provides specialist care to many pregnant women within and beyond South-west Nigeria. On a monthly basis, the hospital has an average of 875 pregnant women attending antenatal clinic, 160 deliveries, and 65 women attend postnatal clinic.

Parturients who were delivered of their babies in the tertiary hospital where the study was conducted and had received postnatal counselling were recruited on the day of discharge. Counselling was conducted as part of the routine discharge procedures, and the duration varied slightly depending on patient needs and clinical workload. Women who had still-births, those whose babies were admitted in the special care baby unit or had early neonatal death and women who were referred to the hospital after they delivered in an outside facility were excluded from the study. All women were counselled at discharge by the nurses and/or doctors according to the departmental postpartum counselling protocol to ensure consistency in content. This protocol was developed internally by the Obstetrics and Gynaecology department and includes standardized information on postnatal care, warning signs and complications, exclusive breastfeeding, child immunization, family planning options, perineal or caesarean wound care, when and where to seek help if needed, and the scheduling of postnatal follow-up. While not formally published, it has been consistently implemented in our department for several decades. For the purposes of this study, no additional training or study-specific instructions were provided to the healthcare providers; counselling was delivered as part of routine clinical practice, and the time and content were consistent with standard discharge counselling for all postpartum women.

### Sample size and sampling

The minimum sample size for the study was calculated using the formula n = Zα^2^(pq)/d^2^ for descriptive studies where Z = 1.96 for a 95% confidence level, *p* = 0.551 (prevalence from a previous study indicating the proportion of mothers with adequate knowledge of obstetrics complications) [12], q = 1 − *p* = 0.449, and d = 0.05 (margin of error). The required minimum sample size at ensure adequate statistical power was 380 parturients. However, a total of 450 women who gave birth in the hospital and consented to participate during the study period were recruited.

**Fig. 1:**
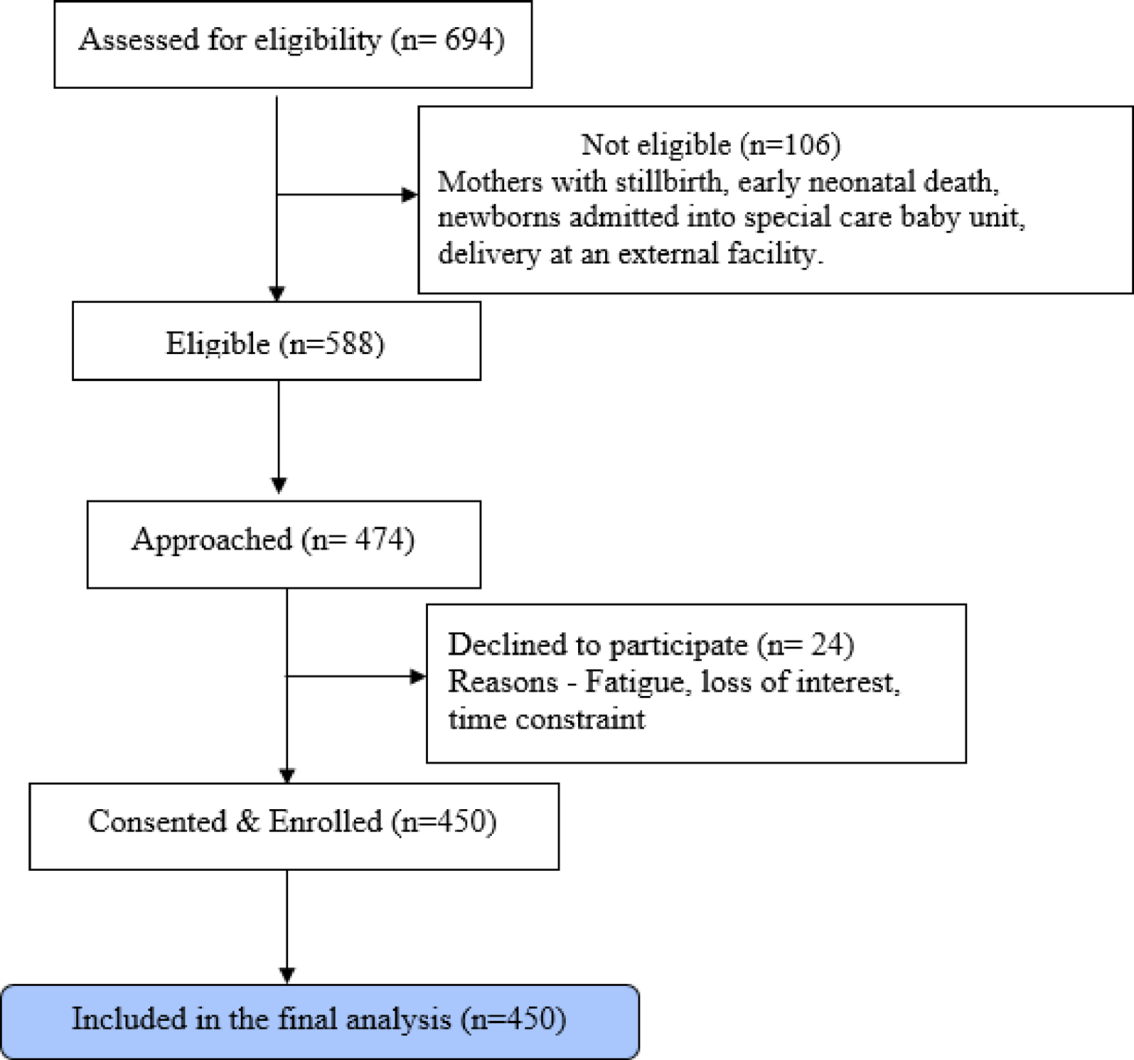
Study flow diagram

The sampling frame comprised all parturients who delivered at the University College Hospital, Ibadan during the study period and met the inclusion criteria. Each day, a list of eligible parturients scheduled for discharge was obtained from the postnatal ward register and reviewed in collaboration with attending nurses. A systematic convenience sampling was used. Eligible parturients were approached consecutively based on the ward discharge scheduling order and the first five consenting eligible women per day were recruited. This was repeated daily till the desired sample size was achieved. To reduce potential bias, participants were recruited across weekdays and weekends and the questionnaire was administered after discharge procedures including counselling were completed on the day of discharge. The refusal rate was low, 5.1% (24 out of the 474 parturients approached), (Fig. 1 – study flowchart).

### Data collection tool

A pre-tested semi-structured self-administered questionnaire was used among 30 parturients to obtain information on knowledge of postnatal warnings signs and complications in a similar setting; Adeoyo Maternity Teaching Hospital, Ibadan prior to the main study to test for clarity, understanding of the questions, and the validity. There was strong internal consistency and good reliability among the variables for the level of knowledge questions as Cronbach alpha was > 0.8.

Questionnaire (Supplementary 1) was developed from WHO postpartum care manual and previous studies [18–20]. The questionnaire included five sections: sociodemographic characteristics (3 items), obstetrics characteristics (4 items), knowledge of postpartum warning signs (10 items), knowledge of postpartum complications (14 items), and related components of postpartum counselling (3 items).

The total knowledge scores were computed, with one point given to a correct response and no point given to the incorrect response. A parturient was assessed to have adequate knowledge if she correctly identified at least seven of the fourteen postpartum complications listed and five postpartum warning signs. The above method of scoring has been previously used by studies conducted to assess knowledge of obstetric danger signs [21, 22].

### Statistical analysis

Data obtained was analysed using IBM SPSS Statistics for Windows, version 25.0 (IBM Corp., Armonk, NY, USA). Descriptive statistics such as: mean, standard deviation, frequency, percentages, and charts was used to summarize the result. The degree of association between the independent and dependent variables was analysed using odds ratios with 95% confidence intervals. The dependent variable was the parturients’ knowledge of postpartum complications dichotomized as adequate = 1 and inadequate = 0. The independent variables included sociodemographic characteristics (age, marital status, level of education), obstetric history (parity, mode of delivery, number of living children), exposure-related variables (timing and duration of counselling and duration of hospital stay after delivery). Multivariate analysis was performed using binary logistic regression to determine the independent determinants of adequate knowledge of postpartum complications among the parturients. Variables that were significant at *p* < 0.05 in the bivariate analysis were included in the logistic regression model. Odds ratios (ORs) with 95% confidence interval were reported and statistical significance was set at *p* < 0.05. The model fitness was checked using Hosmer-Lemeshow goodness-of-fit test.

### Operational definition of terms

For the purpose of this study, parturients are defined as women who have recently given birth and are in the early postpartum period (from 24 h to 7 days). Postpartum period refers to the time after delivery of the new-born till six weeks (42 days). Warning signs/danger signs refer to symptoms that indicates a potential postpartum health risk or presence of condition that increases the chances of a parturient dying or having poor postpartum health state while complications refer to pregnancy related abnormality or health problems that are experienced by the postpartum mother, their baby, or both after delivery. Knowledge was assessed based on participants correct identification of key warning signs and complications. Being knowledgeable means having the basic average information on postpartum warning signs or complications for which the parturient respond correctly to knowledge questions. In addition, the duration of counselling on postpartum warning signs and complications was categorized into *≤* 10 min, 11 to 20 min, 21 to 30 min, and greater than 30 min. Counselling was provided at different time points during the hospital stay, including: (i) throughout the postpartum hospital stay, (ii) only on the day of discharge, (iii) both during the hospital stay and on the day of discharge [23].

## Results

### Basic characteristics of the parturients

A total of 450 parturients participated in the study. The mean age of parturients was 30.1(SD *±* 4.9) years. On the day of discharge 46.9% reported having an excellent overall health, while only 2.4% reported poor overall health. Majority had tertiary level of education (79.8%) and equal proportion (54.9%) of them were both primipara and had spontaneous vaginal delivery (Table 1).

**Table 1:** Parturients’ characteristics

Variable	Frequency (%) *n* = 450	95% CI
Age (years) Mean ± SD - (30.1 ± 4.9)		29.618–30.533
< 25	56 (12.4)	
25–29	150 (33.3)	
30–34	153 (34.0)	
≥ 35	91 (20.2)	
Marital status		0.555–2.784
Single	28 (6.2)	
Married	420 (93.3)	
Divorced/Separated	2 (0.4)	
Highest level of education		0.212–0.705
Secondary/lower	91 (20.2)	
Tertiary/higher	359 (79.8)	
Perceived overall health		1.659–1.808
Excellent	211 (46.9)	
Good	159 (35.3)	
Fair	69 (15.3)	
Poor	11 (2.4)	
Parity Mean ± SD - (1.70 ± 100)		1.417–1.516
1	247 (54.9)	
2–4	196 (43.6)	
*≥* 5	7 (1.6)	
Number of living children		1.513–1.683
One	262 (58.2)	
Two or more	188 (41.8)	
Mode of delivery		0.511–1.116
Spontaneous Vaginal Delivery (SVD)	247 (54.9)	
Caesarean Section (CS)	203 (45.1)	

### Postpartum counselling

Of the parturients, 41.5% were counselled between 11 and 20 min. About four-fifth (81.9%) of the parturients agreed the time allotted for counselling was appropriate with 40.9% of them counselled on the day of discharge only. Over half (54.4%) of them were discharged between the third and fourth day after delivery (Table 2).

**Table 2:** Postpartum counselling among the parturients

Variable	Frequency (%)	95% CI
Duration of Counselling (minutes)		1.731–1.896
≤ 10	139 (36.0)	
11–20	160 (41.5)	
21–30	60 (15.5)	
> 30	27 (7.0)	
Do you think the Counselling time was appropriate?		0.991–1.089
Yes	316 (81.9)	
No	70 (18.1)	
Counselling Period		1.856–1.989
Throughout stay in the hospital only	129 (33.4)	
On the day of discharge only	158 (40.9)	
Throughout stay in the hospital and at discharge	99 (25.7)	
Duration of hospital stay after delivery (days)		2.154–2.273
≤ 2	46 (11.9)	
3–4	210 (54.4)	
≥ 5	130 (33.7)	

### **Parturients’ knowledge of postpartum warning signs**

Among the 180 parturients with the knowledge of warning signs, almost all (99.4%) knew one warning sign, 75.6% knew two warning signs though only 11.7% knew five warning signs (Table 3). The most frequently reported postpartum warning signs among the parturients were foul-smelling lochia 100 (55.6%), bleeding per vagina 91 (50.6%), pain (including abdominal pain, painful breast engorgement, or cracked nipples) 74 (41.1%), and high blood pressure 58(32.2%). Less commonly reported signs included fever 29 (16.1%) and convulsions 39 (21.7%). Other warning signs identified by a smaller proportion of parturients included oedema, breathing difficulties, headache, and thoughts of harming oneself or the baby.

**Table 3:** Parturients knowledge of postpartum warning signs

Knowledge of warning signs	Frequency (%)	Mean ± SD	95% CI
Knowledge of at least a sign	179 (99.4%)	3.28 ± 1.87	3.008–3.559
Knowledge of two signs	136 (75.6%)	4.48 ± 2.05	4.176–4.779
Knowledge of three signs	97 (53.9%)	4.27 ± 1.39	4.068–4.477
Knowledge of four signs	43 (23.9%)	4.63 ± 1.12	4.463–4.792
Knowledge of five signs	21 (11.7%)	4.89 ± 0.44	4.792–4.986

### **Parturients’ knowledge of postpartum complications**

**Fig. 2:**
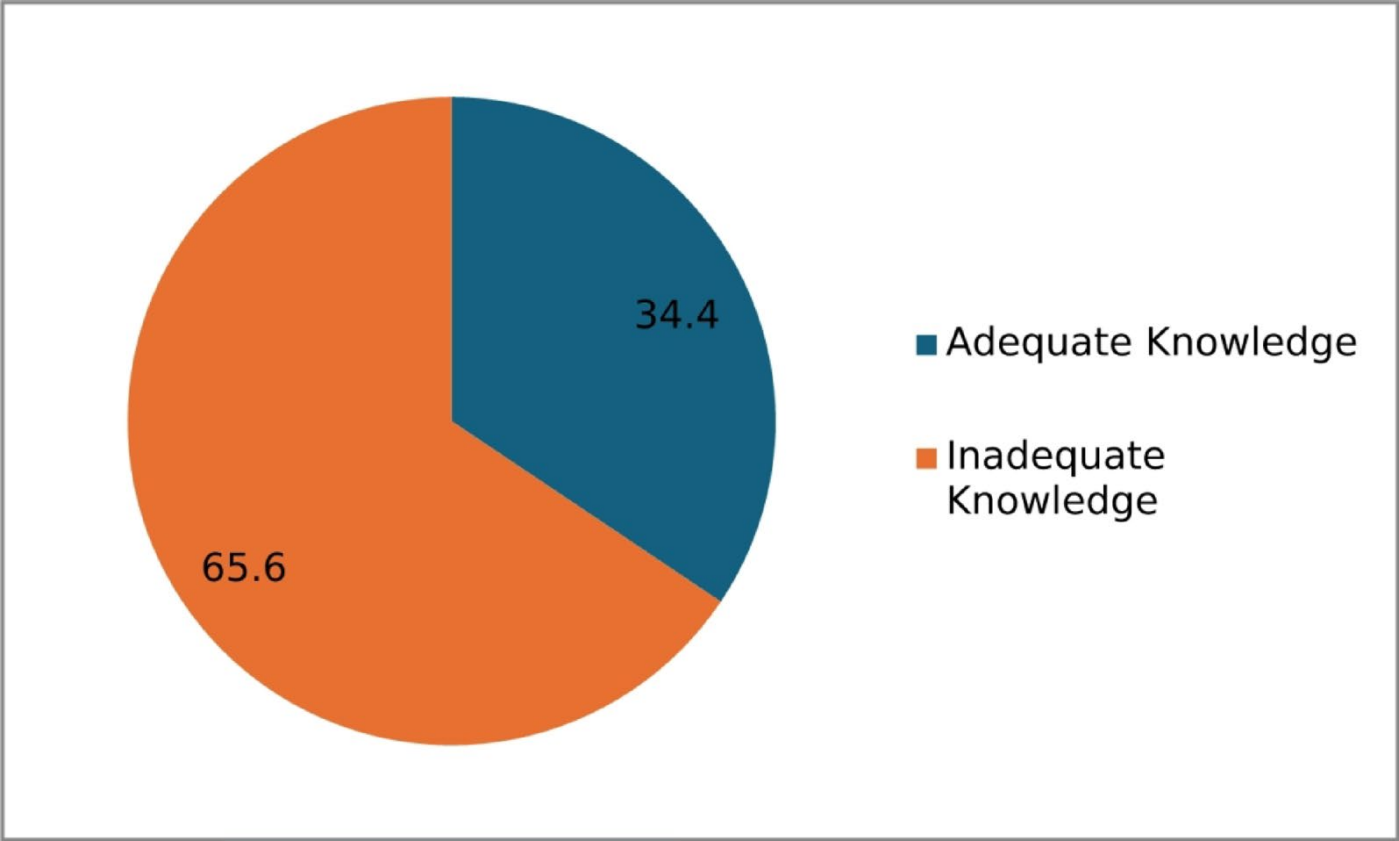
Respondents’ knowledge of postpartum complications

Of the 14 complications listed, the most common complications known among the mothers were wound infection (29.3%), infection/sepsis (25.8%), episiotomy pain (22.9%), and pregnancy-induced hypertension (22.7%) (Table 4). Only 34.4% of the parturients have adequate knowledge of postpartum complications, with a mean of 1.94 (SD = 2.87), 95% CI = 1.669–2.202 (Fig. 2).

**Table 4:** Parturients knowledge of postpartum complications

Variable	Yes (%)	No (%)	Can’t remember (%)	Mean ± SD	95% CI
Knowledge of postpartum complications					
Postpartum haemorrhage	98 (21.8)	348 (77.3)	4 (0.9)	1.79 ± 0.42	1.751–1.831
Postpartum depression	53 (11.8)	391 (86.9)	6 (1.3)	1.896 ± 0.35	1.863–1.928
Infection/sepsis	116 (25.8)	331 (73.6)	3 (0.7)	1.75 ± 0.45	1.707–1.791
Pulmonary embolism	11 (2.4)	414 (92.0)	25 (5.6)	2.03 ± 0.28	2.005–2.057
Pregnancy-induced Hypertension	102 (22.7)	346 (76.9)	2 (0.4)	1.78 ± 0.43	1.738–1.817
Preeclampsia/eclampsia	56 (12.4)	384 (85.3)	10 (2.2)	1.89 ± 0.37	1.864–1.932
Cardiac event	34 (7.6)	405 (90.0)	11 (2.4)	1.95 ± 0.31	1.920–1.978
Venous thrombosis	10 (2.2)	424 (94.2)	16 (3.6)	2.01 ± 0.24	1.991–2.036
Lochia abnormalities	39 (8.7)	396 (88.0)	15 (3.3)	1.95 ± 0.34	1.915–1.978
Mastitis	67 (14.9)	374 (83.1)	9 (2.0)	1.87 ± 0.39	1.835–1.907
Endometritis symptoms	36 (8.0)	400 (88.9)	14 (3.1)	1.95 ± 0.33	1.921–1.982
Uterine prolapse	14 (3.1)	419 (93.1)	17 (3.8)	1.99 ± 0.29	1.971–2.025
Episiotomy pain	103 (22.9)	335 (74.4)	12 (2.7)	1.74 ± 0.55	1.694–1.795
Wound infection	132 (29.3)	309 (68.7)	9 (2.0)	1.60 ± 0.64	1.543–1.661

### Factors associated with knowledge of postpartum complications

In the bivariate analysis, age, education level, and duration of counselling were significantly associated with knowledge of postpartum complications. Parturients aged 25–29 years had the highest proportion of adequate knowledge (42.0%), while those < 25 years had the lowest proportion of adequate knowledge (16.1%) (*p* = 0.003). Only 19.0% of women with secondary or lower education had adequate knowledge compared to 37.7% of those with tertiary or higher education (*p* = 0.001). Furthermore, less than a fifth (18.5%) of parturients who received counselling for less than 10 min had adequate knowledge, compared to 81.5% of those who received counselling for more than 30 min (*p* < 0.001) (Table 5).

**Table 5:** Bivariate analysis of parturients’ characteristics on their knowledge of postpartum complications

Variables	Knowledge of postpartum complications		Chi-square	*P*-value	Odds ratio (OR)
	Adequate	Inadequate			
Age			14.09	0.003	
< 25	9 (16.1)	47 (83.9)			0.83
25–29	63 (42.0)	87 (58.0)			**1.81**
30–34	57 (37.3)	96 (62.7)			1.48
≥ 35	26 (28.6)	65 (71.4)			
Marital status			0.28	0.596	0.63
Married	146 (34.8)	274 (65.2)			
Others (ref)	9 (30.0)	21 (70.0)			
Highest level of education			**10.14**	**0.001**	
Secondary/lower	15 (19.0)	64 (81.0)			**0.43**
Tertiary/higher (ref)	140 (37.7)	231 (62.3)			
Mode of delivery			1.99	0.158	
SVD	78 (31.6)	169 (68.4)			0.76
CS (ref)	77 (37.9)	126 (62.1)			
Parity			4.04	0.132	
1	75 (30.4)	172 (69.6)			0.56
2	47 (39.2)	73 (60.8)			0.72
≥ 3 (ref)	33 (39.8)	50 (60.2)			
Duration of time spent on counselling (minutes)			**57.37**	**< 0.001**	
< 10	37 (18.5)	163 (81.5)			**0.05**
11–20	66 (41.0)	95 (59.0)			0.16
21–30	30 (48.4)	32 (51.6)			0.23
> 30 (ref)	22 (81.5)	5 (18.5)			
Duration of hospital stay after delivery (days)			1.25	0.537	
≤ 2	16 (28.6)	40 (71.4)			0.35
3–4	83 (34.2)	159 (65.7)			0.96
≥ 5 (ref)	56 (36.8)	96 (63.2)			

Bold values are statistically significant (*p* < 0.05)

### Factors determining the parturients knowledge of postpartum complications

Parturients aged 25–29 years were twice more likely to be knowledgeable of postpartum complications compared to those ≥ 35 year (p value = 0.026, OR = 2.37, 95% CI  = 1.107–5.057). Those with secondary/lower level of education were thrice less likely to be knowledgeable of postpartum complications compared with those with tertiary/higher level of education (p value = 0.001, OR = 0.37, 95% CI = 0.208–0.650).

Additionally, parturients who received information on postpartum complications for less than 10 min were about 8 times less likely to be knowledgeable on postpartum complications compared to those who received information on postpartum complications for > 30 min (p value = 0.001, OR = 0.13, 95% CI = 0.041–0.425), (Table 6).

**Table 6:** Determinants of parturients’ knowledge of postpartum complications

Variables	Odds ratio	*p*-value	95% CI
Age			
< 25	0.98	0.978	0.298–3.245
25–29	**2.37**	**0.026**	**1.107–5.057**
30–34	1.67	0.161	0.814–3.435
≥ 35 (ref)	1	-	-
Highest level of education			
Secondary/lower	**0.37**	**0.001**	**0.208–0.650**
Tertiary/higher(ref)	1	-	-
Duration of time spent on counselling (minutes)			
< 10	**0.13**	**0.001**	**0.041–0.425**
11–20	0.33	0.057	0.107–1.034
21–30	0.29	0.051	0.085–1.003
> 30(ref)	1	-	-

Bold values are statistically significant (*p* < 0.05)

## Discussion

The overall knowledge of postpartum warning signs and complications among the parturients was low. Age, educational status, and duration of time used to relay information were determinants of parturients’ knowledge of postpartum complications and are crucial to developing interventions in preventing postpartum complications with resultant maternal morbidities and mortalities.

About a third of the parturients were observed to have good knowledge of postpartum complications. Studies have also reported similar findings of low level of knowledge among same group of postnatal women [12, 24]. This could be that the mothers received insufficient information on this topic due to poor communication or lack of up-to-date information regarding maternal morbidity and mortality after childbirth among the healthcare workers thus they might not be able to provide comprehensive education about postpartum care, warning signs, and complications to the new mothers before they were discharged from the hospital [19, 23, 25].

The main postpartum complications known by the parturients were wound infection, infection/sepsis, episiotomy pain, and hypertension. Among these wound infection and infection/sepsis were the most reported. This may be attributed to the visible and distressing nature of wound infections, as well as the higher incidence of infection in low-resource settings, where many infections-related maternal deaths remain preventable [26]. These findings are consistent with some of the known leading causes of maternal morbidity and deaths in Nigeria [9–11]. A mixed methods study on midwives’ perceptions of maternal mortality in Nigeria identified postpartum haemorrhage, hypertensive disorders, and sepsis as the leading causes, with mismanagement by traditional birth attendants and delays in care-seeking as major external contributors [27]. Additionally, studies have linked the level of knowledge and competency regarding postpartum warning signs and complications among midwives to the quality of health education given to women [18, 19, 23, 26, 27]. A quasi-experimental study among Nigerian midwives revealed only 26.4% had a good knowledge of postpartum warning signs but this improved significantly to 95.4% after undergoing training on postpartum warning signs [25]. Similarly, a cross-sectional study in Ghana on knowledge and teaching of postpartum complications among midwives’ reported haemorrhage, infection, preeclampsia/eclampsia, and hypertension as the four main complications taught because of their competency in these areas compared to other known complications [28]. This highlights how midwives’ knowledge influences the quality of postpartum education provided to mothers on the identification and recognition of warning signs and complications, which may have contributed to the low levels of knowledge observed among parturients in our study.

In addition, almost all the parturients knew at least a warning sign of postpartum complication but less than one-sixth knew five danger signs. Similarly, a study on knowledge of postnatal care among postnatal mothers reported a gradual decrease in proportion of knowledge of at least a danger sign to knowing a fifth danger sign [29]. Studies have also shown poor knowledge of complication readiness and obstetric danger signs during postpartum period among antenatal attendees and married men [22, 30, 31].

Foul smelling lochia, bleeding, and pain were the common signs reported. This finding is in keeping with the findings from other studies [29, 32]. In a study that determined the knowledge on birth preparedness and complication readiness and associated factors among primigravidae, bleeding was the most reported warning sign during the postpartum period [32]. Another study that assessed the knowledge on postnatal care among postnatal mothers, heavy bleeding, postpartum eclampsia, and puerperal infection were the identified danger signs of postpartum complications [29]. Postpartum haemorrhage is the leading cause of maternal mortality in sub-Saharan Africa, and it was also the most reported severe maternal morbidity outcome resulting in 123 maternal deaths and 251 near-misses in 42 Nigerian tertiary facilities in a year [5, 33, 34]. This is corroborated by our finding of postpartum bleeding as the second commonest warning sign of postpartum complication known by the parturients. On the other hand, a study among pregnant women in Ethiopia reported blurred vision as the most frequently known postpartum danger sign with 16.8% overall level of good knowledge which was lower than the findings in this study [14]. The possible reason might be the different study population, non-uniformity of health education materials, inconsistencies in counselling approaches, and practices across healthcare facilities.

The determinants of knowledge of postpartum complications were age, education, and duration of time used in counselling. Younger parturients are more likely to be knowledgeable. Similar observation was shown among antenatal attendees in Ethiopia [35]. This finding could be because most of the younger age women are new mothers and are likely to give more attention to details of postpartum counselling since they do not have prior experience.

Education is essential for easy understanding of health-related issues including mothers’ autonomy to make decisions regarding their reproductive and healthcare utilization due to their increased awareness about their health conditions. Studies have shown a linear and positive association between health literacy and educational attainment [36, 37]. A preponderance of the parturients in our study has high educational level and depicted those with least educational status as not having adequate knowledge of postpartum complications. Similarly, a study on knowledge about danger signs of obstetric complications and associated factors among postnatal mothers in Ethiopia revealed mothers with higher level of education to be more knowledgeable [12].

The duration of counselling was another determinant of being knowledgeable of postpartum complications in the index study. The majority of the parturients were counselled for 11–20 min and those who received counselling for a shorter duration were significantly less likely to be knowledgeable about postpartum complications. Surprisingly, majority of them (4 in 5 of the parturients) agreed the time spent at counselling or teaching postpartum complications was appropriate. This finding is not unexpected because it is normal that the more time spent at relaying salient points, the more it is readily and easily absorbed by the audience. To buttress this, a survey of registered nurses in the United States revealed that above two-thirds of the nurses taught the potential warning signs of postpartum complications to the new mothers for less than 10 min on the day of discharge [19]. Counselling duration may serve as a proxy for the quality of postpartum education, as longer sessions may allow for better engagement and individualized support. However, the effectiveness of counselling does not only depend on time spent but also on how that time is used. Studies have documented the importance and effectiveness of using structured discharge protocols, such as standardized postpartum checklists that cover warning signs, complications, and care instructions [20, 38]. In addition, involving partners or family members in counselling sessions may improve support systems and reinforce health-seeking behaviours after discharge [22, 38].

It is worthy to note that factors such as parity, gravidity, and marital status were not predictors of the knowledge of postpartum complications, unlike other studies that reported correlation between these factors with knowledge of postpartum care or complications [12, 32].

This study used a detailed questionnaire adapted from the WHO postpartum care and complications and previous studies to identify the knowledge of the parturients. The findings from this study provide information on the determinants of adequate knowledge of postpartum warning signs and complications among postpartum mothers. This can be utilized in developing strategies to provide effective counselling and address the components and timing of counselling about postpartum warning signs and complications which can improve parturients’ knowledge.

This study is not without its limitation, as a tertiary hospital-based study involving predominantly well-educated women, the findings may not be generalized to women who had home delivery, delivered by traditional birth attendants, or received care in secondary and primary health facilities. Thus, the study should be interpreted with caution in relation to educational status and healthcare access. Additionally, information on the number of antenatal care visits was not explored as this could have revealed the correlation between the frequencies of visit with the level of knowledge since the women could have gotten some information on postpartum care, warning signs, and complications during antenatal care visit. Variations in the content and delivery format of postpartum counselling were also not assessed, whether counselling was delivered by a nurse or doctor. Although both cadres routinely provide counselling in the study setting, the lack of specific measurement of the provider or format may have introduced unmeasured confounding. While the use of a self-administered questionnaire helped to reduce interviewer bias, social desirability bias may still have influenced participants’ responses, given the hospital setting and recent counselling. However, the relatively low proportion of women with adequate knowledge suggests that the impact of social desirability bias on the findings was likely minimal. Future research should include a more diverse women population to enhance generalizability and explore the influence of antenatal clinic attendance which could provide valuable insights into the role of antenatal care in maternal education. Additionally, longitudinal studies are needed to explore the association between parturient knowledge of postpartum warning signs and complications with the time of presentation for medical intervention among those who develop postpartum complications.

## Conclusions

The knowledge of postpartum complications among the parturients is low despite exposure to postpartum counselling. Parturient age, educational level, and the duration of counselling were significantly associated with knowledge levels. There is a need to improve postpartum counselling/education during hospital stay and at discharge to ensure women are adequately informed about warning signs and complications, thereby empowering them to recognise symptoms early and seek prompt care. To address this gap, information on postpartum warning signs and complications should be delivered to all parturient in a standardized manner and for a standard average time irrespective of their parity. Healthcare providers should implement targeted programs and use tailored information, education, and communication materials and postpartum checklists to increase awareness and knowledge of postpartum warning signs and complications.

## Supplementary Information

Below is the link to the electronic supplementary material.

Supplementary Material 1

Supplementary Material 2

Supplementary Material 3

Supplementary Material 4

Supplementary Material 5

Supplementary Material 6

Supplementary Material 7

## Data Availability

Data is provided within the supplementary information files.

## Abbreviations

SVD: Spontaneous Vaginal Delivery
CS: Caesarean Section
MMR: Maternal Mortality Ratio
SDG: Sustainable Development Goals
