# Supplementary material for: Determinants of adequate knowledge of postpartum warning signs and complications among parturients in Ibadan, Nigeria: a cross sectional study
Source: BMC Pregnancy Childbirth. 2025 Aug 28;25:894. doi: 10.1186/s12884-025-08058-1 (PMC12392526; doi:10.1186/s12884-025-08058-1)
Supplement: Supplementary file 2 — Supplementary Material 2 [file 12884_2025_8058_MOESM2_ESM.docx]

**Determinants of Adequate Knowledge of Postpartum Warning Signs and Complications Among Parturients in Ibadan, Nigeria: A Cross sectional study.**

**STROBE Checklist**

| **Item No** | **Recommendation** | **Page Number & line** |
| --- | --- | --- |
| **Title and Abstract** | | |
| 1. | a. Indicate the study's design with a commonly used term in the title or the abstract.  b. Provide in the abstract an informative and balanced summary of what was done and what was found. | Page 1-2, lines 2, 17  Page 1-2, lines 12-36 |
| **Introduction** | |  |
| 2. | Background/Rationale: Explain the scientific background and rationale for the investigation being reported. | Page 2-5, lines 39-104 |
| 3. | Objectives: State specific objectives, including any prespecified hypotheses. | Page 5, lines 102-104 |
| **Methods** | | |
| 4. | Study design: Present key elements of study design early in the paper. | Page 5, lines 108 |
| 5. | Settings: Describe the setting, locations, and relevant dates, including periods of recruitment, exposure, follow-up, and data collection. | Page 5-7, lines 110-133; 153-166 |
| 6. | Participants: Describe eligibility criteria and the sources and methods of selection of participants. Describe methods of follow-up. | Page 5-6, lines 118-125, Page 7, lines 142-148 |
| 7. | Variables: Clearly define all outcomes, exposures, predictors, potential confounders, and effect modifiers. | Page 8, lines 171-183 |
| 8. | Data sources/measurement: For each variable of interest, give sources of data and details of methods of assessment (measurement). | Page 7-8, lines 157-166, 184-198 |
| 9. | Bias: Describe any efforts to address potential sources of bias. | Page 7, lines 148-150 |
| 10. | Study size: Explain how the study size was arrived at. | Page 6, lines 135-141 |
| 11. | Quantitative variables: Explain how quantitative variables were handled in the analyses. If applicable, describe which groupings were chosen and why. | Page 8, lines 169-183 |
| 12. | a. Statistical methods: Describe all statistical methods, including those used to control for confounding.  b. Describe any methods used to examine subgroups and interactions.  c. Explain how missing data were addressed.  d. If applicable, explain how loss to follow-up was addressed.  e. Describe any sensitivity analyses. | Page 8, lines 170-173  Page, lines 178-183  For variables with missing responses, valid percentages were reported by excluding cases with missing data from the denominator.  Not applicable  Not applicable |
| **Results** | | |
| 13. | a. Participants: Report numbers of individuals at each stage of study—e.g., numbers potentially eligible, examined for eligibility, confirmed eligible, included in the study, completing follow-up, and analysed.  b. Give reasons for non-participants at each stage.  c. Consider use of a flow diagram | Page 9, lines 209-228  Page 9, lines 209-228  Page 9, line 199 |
| 14. | Descriptive data: Give characteristics of study participants (e.g., demographic, clinical, social) and information on exposures and potential confounders. | Page 10&11, lines 232-240 |
| 15. | Outcome data: Report numbers of outcome events or summary measures over time. | Page 10-17, lines 232-301 |
| 16. | Main results: Give unadjusted estimates and, if applicable, confounder-adjusted estimates and their precision (e.g., 95% confidence interval). Make clear which confounders were adjusted for and why they were included. | Page 15-17, lines 276-301 |
| 17. | Other analyses: Report other analyses done e.g., analyses of subgroups and interactions, and sensitivity analyses. | Page 15-17, lines 276-282 |
| **Discussion** | | |
| 18. | Key results: Summarize key results with reference to study objectives. | Page 18, lines 303-307 |
| 19. | Limitations: Discuss limitations of the study, taking into account sources of potential bias or imprecision. Discuss both direction and magnitude of any potential bias. | Page 22-23, lines 402-417 |
| 20. | Interpretation: Give a cautious overall interpretation of results considering objectives, limitations, multiplicity of analyses, results from similar studies, and other relevant evidence. | Page 18-21, lines 302-401 |
| 21. | Generalizability: Discuss the generalisability (external validity) of the study results. | Page 21, lines 402-406 |
| **Other information** | | |
| 22. | Funding: Give the source of funding and the role of the funders for the present study and, if applicable, for the original study on which the present article is based. | Page 24, line 456 |

The STROBE checklist used in this study follows the guidelines outlined by von Elm et al. (2007), titled The Strengthening the Reporting of Observational Studies in Epidemiology (STROBE) Statement: Guidelines for Reporting Observational Studies, published in the Annals of Internal Medicine, 147(8), 573–577. <https://doi.org/10.7326/0003-4819-147-8-200710160-00010>
