# Supplementary material for: Determinants of adequate knowledge of postpartum warning signs and complications among parturients in Ibadan, Nigeria: a cross sectional study
Source: BMC Pregnancy Childbirth. 2025 Aug 28;25:894. doi: 10.1186/s12884-025-08058-1 (PMC12392526; doi:10.1186/s12884-025-08058-1)
Supplement: Supplementary file 3 — Supplementary Material 3 [file 12884_2025_8058_MOESM3_ESM.docx]

**Means, standard deviations (SD) and Cronbach’s Alpha values of the Postpartum complication variables**

| **Scales** | **Mean + SD** | **Cronbach's alpha** |
| --- | --- | --- |
| Postpartum haemorrhage | 1.77 + 0.57 | 0.79 |
| Postpartum depression | 1.90 + 0.40 | 0.79 |
| Infection/Sepsis | 1.53 + 0.57 | 0.80 |
| Pulmonary embolism | 2.13 + 0.51 | 0.83 |
| Pregnancy-Induced Hypertension | 1.60 + 0.50 | 0.82 |
| Preeclampsia/Eclampsia | 1.70 + 0.53 | 0.81 |
| Cardiac event | 1.87 + 0.43 | 0.79 |
| Venous thrombosis | 2.07 + 0.37 | 0.80 |
| Lochia abnormalities | 2.10 + 0.48 | 0.80 |
| Mastitis | 1.90 + 0.55 | 0.79 |
| Endometritis symptoms/signs | 2.01 + 0.45 | 0.80 |
| Uterine Prolapse | 2.03 + 0.41 | 0.80 |
| Episiotomy pain *(those with episiotomy ONLY)* | 1.70 + 0.70 | 0.80 |
| Wound Infection *(those with C/S ONLY)* | 1.4 + 0.56 | 0.81 |

**Logistic Regression Model Diagnostics**

| **Diagnostic Metric** | **Result** | **Interpretation** |
| --- | --- | --- |
| Likelihood Ratio Chi-square (χ²) | 80.47, df = 13, p < 0.001 | Model is statistically significant |
| Hosmer–Lemeshow Test | χ² = 8.24, df = 8, p = 0.410 | Good model fit (p > 0.05) |
| ROC-AUC | 0.742 | Acceptable discrimination (74.2% accuracy) |
| Variance Inflation Factor (VIF) | All VIFs < 2 | No multicollinearity among independent variables |

**Collinearity check**

| **Predictor Variable** | **Tolerance** | **VIF** |
| --- | --- | --- |
| Age recoded into 4 categories | 0.672 | 1.487 |
| Marital Status in 2 | 0.893 | 1.119 |
| Education Level in 2 | 0.949 | 1.054 |
| Mode of Delivery in 2 | 0.640 | 1.563 |
| Parity in 3 | 0.734 | 1.363 |
| Duration of Counselling in 4 | 0.976 | 1.024 |
| Duration of Hospital Stay in 3 | 0.648 | 1.544 |
