# Supplementary material for: Determinants of adequate knowledge of postpartum warning signs and complications among parturients in Ibadan, Nigeria: a cross sectional study
Source: BMC Pregnancy Childbirth. 2025 Aug 28;25:894. doi: 10.1186/s12884-025-08058-1 (PMC12392526; doi:10.1186/s12884-025-08058-1)
Supplement: Supplementary file 7 — Supplementary Material 7 [file 12884_2025_8058_MOESM7_ESM.docx]

**Determinants of Adequate Knowledge of Postpartum Warning Signs and Complications Among Parturients in Ibadan, Nigeria: A Cross-sectional study**

**SECTION A: SOCIO-DEMOGRAPHIC CHARACTERISTICS**

1. Age………… years
2. Marital status:
3. Single ☐
4. Married ☐
5. Separated ☐
6. Divorced ☐
7. Highest level of education:
8. None ☐
9. Primary ☐
10. Secondary ☐
11. Tertiary ☐

**SECTION B: OBSTETRICS & POSTPARTUM CHARACTERISTICS**

1. How many times have you given birth (after 28 weeks/7 months of pregnancy)? (a) Number of births……………

(b) Number of living children…………

1. Mode of delivery:

(a) Vaginal delivery ☐

(b) Caesarean section ☐

(c) Instrumental Vaginal delivery (e.g., Forceps or Vacuum) ☐

1. Overall, how would you rate your current health?
2. Excellent ☐
3. Very Good ☐
4. Good ☐
5. Fair ☐
6. Poor ☐
7. How long did you stay in the hospital after delivery?
8. 1 day ☐
9. 2 days ☐
10. 3 days ☐
11. 4 days ☐
12. 5 days ☐
13. > 5 days ☐

**SECTION C:** **KNOWLEDGE OF POSTPARTUM WARNING SIGNS**

1. List any postpartum warning signs or danger signs you know or were told about during your counselling or health education after delivery?
2. ………………………………………
3. ………………………………………
4. ………………………………………
5. ………………………………………
6. ………………………………………
7. ………………………………………
8. ………………………………………
9. ………………………………………
10. ………………………………………
11. ………………………………………

**SECTION D:** **KNOWLEDGE ABOUT POSTPARTUM COMPLICATIONS**

1. Which of the following postpartum complications were discussed with you during your counselling or health education after delivery? (Please select one response for each item)?

| **COMPLICATIONS** | **YES** | **NO** | **CAN’T REMEMBER** |
| --- | --- | --- | --- |
| Postpartum haemorrhage |  |  |  |
| Postpartum depression |  |  |  |
| Infection/Sepsis |  |  |  |
| Pulmonary embolism |  |  |  |
| Hypertension |  |  |  |
| Preeclampsia/Eclampsia |  |  |  |
| Cardiac event |  |  |  |
| Venous thrombosis |  |  |  |
| Lochia abnormalities |  |  |  |
| Mastitis |  |  |  |
| Endometritis symptoms/signs |  |  |  |
| Uterine Prolapse |  |  |  |
| Episiotomy pain |  |  |  |
| Wound Infection |  |  |  |

**SECTION E:** **COMPONENT POSTPARTUM COUNSELLING**

1. Approximately how many minutes were spent counselling you on postpartum complication and warning signs?

(a) < 10 mins ☐

(b) 11-20 mins ☐

(c) 21-30 mins ☐

(d) >30 mins ☐

1. Do you think the amount of time spent on postpartum counselling/education was adequate and appropriate?

(a) Yes ☐

(b) No ☐

1. When did you receive counselling or education on postpartum complications?

(a) Throughout admission ☐

(b) At discharge ☐

(c) Throughout admission and at discharge ☐
